# Nomogram to predict postoperative cognitive dysfunction in elderly patients undergoing gastrointestinal tumor resection

**DOI:** 10.3389/fnagi.2022.1037852

**Published:** 2022-10-25

**Authors:** Huifan Huang, Jing Chou, Yongzhong Tang, Wen Ouyang, Xiaoxia Wu, Yuan Le

**Affiliations:** ^1^Department of Anesthesiology, The Third Xiangya Hospital, Central South University, Changsha, China; ^2^Department of Gastrointestinal Surgery, The Third Xiangya Hospital, Central South University, Changsha, China; ^3^Hunan Province Key Laboratory of Brain Homeostasis, The Third Xiangya Hospital, Central South University, Changsha, China; ^4^Department of Nursing, The Third Xiangya Hospital, Central South University, Changsha, China

**Keywords:** postoperative cognitive dysfunction, gastrointestinal tumors, nomogram, prediction model, risk factors

## Abstract

**Objective:**

To establish a nomogram model for the prediction of postoperative cognitive dysfunction (POCD) in elderly patients undergoing gastrointestinal tumor resection.

**Methods:**

A total of 369 elderly patients scheduled for elective gastrointestinal tumor resection under general anesthesia were included. The cognitive function of each participant was assessed by the Mini-Mental State Examination (MMSE) 1 day before surgery and 7 days after surgery for the diagnosis of POCD. According to the results, patients were divided into a POCD group and a non-POCD group. The differences in hospitalization data and examination results between the two groups were compared. A logistic regression model was used to explore the risk factors for POCD in elderly patients undergoing gastrointestinal tumor resection, and a nomogram was then constructed based on these factors. The diagnostic performance of the nomogram was evaluated using the area under the receiver operating characteristic curve (AUROC) and a calibration plot. The clinical usefulness of the nomogram was estimated using decision curve analysis (DCA).

**Results:**

Among the 369 patients undergoing gastrointestinal tumor resection, 79 patients had POCD, with a positive rate of 21.4%. The nomogram model comprised the following variables: age, body mass index (BMI), history of cerebrovascular disease, preoperative white blood cell (WBC) count, preoperative hemoglobin (Hb) level, intra-operative blood loss, and operation time. The model showed good discrimination, with an area under the curve (AUC) of 0.710 (95% CI = 0.645–0.775), and good calibration (Hosmer–Lemeshow test, χ^2^ = 5.133, *p* = 0.274). Internal validation also maintained ideal discrimination and calibration. Decision curves indicated that when the threshold probability was above 0.1, the nomogram achieved more benefit than both the treat-all and treat-none policies.

**Conclusion:**

This scoring system is the first nomogram model developed for the prediction of POCD in elderly patients undergoing gastrointestinal tumor resection. It has good efficacy in the prediction of POCD risk and could provide an important reference for the prevention, management, and treatment of POCD.

## Introduction

In 2019, 700 million people worldwide were over the age of 65 years, and it is reported that the population will increase to 16% by 2050, in which one out of six people will be elderly (United Nations: Department of Economic and Social Affairs, 2021). With the advent of an aging society, the number of elderly patients with age-related diseases that require surgery is increasing. Therefore, long-term postoperative outcomes and healthcare requirements of elderly patients have become challenging in anesthesia (Cao et al., 2018). Postoperative cognitive dysfunction (POCD) is a common postoperative complication in elderly patients (Peden et al., 2021), and it has been reported that the incidence of cognitive impairment in elderly patients who undergo noncardiac surgery is 25.8–41.4% at 1 week after surgery, which is still as high as 9.9–12.7% at 3 months (Moller et al., 1998; Monk et al., 2008). Patients’ memory, social abilities, executive abilities, etc. are mainly affected by POCD (Eckenhoff et al., 2020). It not only prolongs the hospital stay and increases the medical expenses of patients but also reduces their quality of life and self-care abilities after discharge. Moreover, it increases the incidence of postoperative dementia and even increases the mortality of patients (Newman et al., 2007; Murthy et al., 2015), causing a huge burden on patients, their families, and society (Boone et al., 2020).

At present, clinical prevention and treatment measures for POCD mainly include drug therapy, nondrug therapy, or a combination of both. Nondrug measures include adopting minimally invasive surgery, improving the effective perfusion of tissues and organs during surgery (Borsook et al., 2010), strengthening postoperative analgesia (Evered et al., 2021), controlling the depth of anesthesia (O’Brien et al., 2017), and promoting physical exercise in the perioperative period (Rengel et al., 2021; Duan et al., 2022). Drug methods include the use of dexmedetomidine (Su et al., 2016), COX-2 inhibitors (Margraf et al., 2020), statins (Alam et al., 2018), and probiotics during the perioperative period (Wang et al., 2021). These measures could reduce the incidence of POCD to a certain extent, which suggests that the early identification of high-risk patients who may develop POCD and taking preventive measures as soon as possible may be an effective management strategy for POCD (Kotekar et al., 2018).

A more advanced age, a lower education level (Rundshagen, 2014; Needham et al., 2017; Urits et al., 2019; Mahanna-Gabrielli et al., 2020), a history of cerebrovascular accidents (Urits et al., 2019), poor baseline cognitive status (Zietemann et al., 2018), a higher vulnerability index (Susano et al., 2020), and a history of alcohol consumption (Gvozdenović and Antanasković, 2015) are possible risk factors for the occurrence of POCD. The risk prediction model not only focuses on a single indicator but also includes multiple factors influencing the occurrence of the disease. Therefore, it could more quickly and systematically identify and distinguish the groups at high risk of the disease. However, there have been few reports on POCD prediction model research. Therefore, in this study, we performed a multivariate regression analysis with risk factors reported in previous literature that may affect the development of POCD in elderly patients. Then, based on that, we constructed a nomogram to provide a method to identify groups at high risk for POCD.

## Materials and methods

### Patients and clinical data

This is a retrospective case-control study, approved by the Ethics Committee of The Third Xiangya Hospital (ID: 22035) with waived written consent. A total of 369 elderly patients who were scheduled for selective gastrointestinal tumor resection under general anesthesia in The Third Xiangya Hospital from March 2018 to October 2021 were enrolled. The inclusion criteria were as follows: (1) preoperative Mini-Mental State Examination (MMSE) score >24 points; (2) patients over the age of 60 years; and (3) patients scheduled for gastrointestinal tumor resection under general anesthesia. The exclusion criteria were as follows: (1) patients with a history of severe neurological or psychiatric disorders; (2) patients with a history of sedative or antidepressant medication; (3) patients with a severe audio-visual impairment affecting the assessment; and (4) patients diagnosed with delirium by using the Confusion Assessment Method (CAM) before the surgery. Patients’ baseline characteristics, intraoperative information, and postoperative outcomes in our database were all extracted from electronic medical records.

### Cognitive function assessment and postoperative cognitive dysfunction diagnostic criteria

The cognitive function assessments were performed 1 day before surgery and 7 days after surgery (or at discharge) by an experienced anesthesia nurse who was blinded to this protocol. The MMSE was used for assessments. The MMSE score 1 day before surgery was the baseline score, and POCD was diagnosed when the MMSE score decreased by more than 3 points compared with the baseline score (Wang et al., 2021).

### Statistical analysis

All data analyses were performed using SPSS 26.0 software and R version 4.1.2. Continuous data were expressed as mean ± standard deviation (SD) ($\bar{x}\pm s$) and comparisons between groups were expressed by a *t*-test; while categorical data were presented as numbers with percentages and compared using the χ^2^ test or continuity-corrected χ^2^ test. A multivariate logistic regression analysis was performed to further screen the optimal prediction model with the lowest Akaike information criterion (AIC). A nomogram was built using the rms package in R software based on the final results of multivariate logistic regression. The receiver operating characteristic (ROC) curve was conducted to assess the predictive performance of the model. At the same time, the model was internally validated for discrimination and calibration using 1,000 bootstrap resamplings, and the Hosmer–Lemeshow goodness-of-fit test was used to evaluate the fitting degree of the predictive model. Finally, the clinical usefulness of the nomogram was estimated using the decision curve analysis (DCA). All tests were two-tailed, and *p* < 0.05 was considered statistically significant.

## Results

### Comparison of general data and perioperative data between the postoperative cognitive dysfunction group and the non-postoperative cognitive dysfunction group

A total of 369 patients were included in the final analysis, and 69.9% were men. All patients were divided into the POCD group (*n* = 79) and the non-POCD group (*n* = 290) according to the cognitive function assessment results. The incidence of POCD was 21.4% (79/369). Table 1 shows the baseline characteristics and perioperative data of patients who had POCD and those who did not. Compared with the non-POCD group, the patients in the POCD group were older, had a lower body mass index (BMI), and were more likely to have a previous history of cerebrovascular disease (*p* < 0.05). As for perioperative data, the patients who had POCD had a higher frequency of white blood cell (WBC) count >10 × 10^9^/L, hemoglobin (Hb) level <120 g/L, intraoperative blood loss >400 ml, and operation time >8 h (*p* < 0.05, Table 1).

**TABLE 1:** Comparison of general data and perioperative data between the POCD group and the non-POCD group.

Variables	*n* = 369	POCD (*n* = 79)	nPOCD (*n* = 260)	*P-Value*
Age ≥70 years	154	42 (53.2)	112 (38.6)	0.020*
Sex (male, %)	258	48 (60.8)	209 (72.1)	0.053
BMI <18.5 kg/m^2^, *n* (%)	42	15 (19.0)	27 (9.3)	0.016*
Education ≤9 years, *n* (%)	277	59 (74.7)	218 (75.1)	0.929
ASA ≥III grade, *n* (%)	17	5 (6.3)	12 (4.1)	0.410
MMSE score (mean ± SD)	83.61 ± 11.27	27.43 ± 1.47	27.14 ± 1.62	0.153
**History of disease and medication, *n* (%)**				
Hypertension	127	29 (36.7)	98 (33.8)	0.629
Diabetes	46	13 (16.5)	33 (11.4)	0.226
Cardiovascular disease	38	10 (12.7)	28 (9.7)	0.436
Cerebrovascular disease	23	12 (15.2)	11 (3.8)	< 0.001*
Lung disease	78	19 (24.1)	59 (20.34)	0.474
Blood transfusion	20	5 (6.3)	15 (5.1)	0.687
Chemotherapy	17	3 (3.8)	14 (4.8)	0.933
Smoking	180	31 (39.2)	149 (51.4)	0.056
Alcohol consumption	79	14 (17.7)	65 (22.4)	0.367
**Laboratory tests, *n* (%)**				
WBC >10 (×10^9^/L)	14	8 (10.1)	6 (2.1)	0.001*
Hb >120 g/L	171	46 (58.2)	125 (43.1)	0.017*
Abl <30 g/L	10	1 (1.3)	9 (3.1)	0.616
Cr >133 μmol/L	9	1 (1.3)	8 (2.8)	0.725
NC (mean ± SD)	64.08 ± 10.64	65.43 ± 10.76	63.71 ± 10.59	0.200
LC (mean ± SD)	24.99 ± 9.48	24.67 ± 11.01	25.07 ± 9.03	0.740
NLR (mean ± SD)	3.29 ± 2.61	3.42 ± 2.27	3.25 ± 2.70	0.603
**Operation information, *n* (%)**				
Dexamethasone	88	18 (22.8)	70 (24.1)	0.802
Dexmedetomidine	115	26 (32.9)	89 (30.7)	0.705
Vasopressor drugs	93	25 (31.6)	68 (25.2)	0.138
TAP	194	46 (58.2)	148 (51.0)	0.256
Warm treatment	262	53 (67.1)	209 (72.1)	0.387
Infusion volume	2,964.70 ± 864.56	3,062.99 ± 887.42	2,938.42 ± 858.01	0.262
Urine volume	1,042.11 ± 578.02	1,134.62 ± 632.63	1,016.07 ± 562.96	0.111
Blood loss ≥400 ml	28	12 (15.2)	16 (5.5)	0.004*
Operation time >8 h	9	5 (6.3)	4 (1.4)	0.034*

POCD, postoperative cognitive dysfunction; BMI, body mass index; ASA, American Society of Anesthesiologists; MMSE, Mini-Mental State Examination; WBC, white blood cell; Hb, hemoglobin; Alb, albumin; Cr, creatinine; NC, Neutrophil cell; LC, lymphocyte; NLR, neutrophil-lymphocyte ratio; TAP, transversus abdominis plane block. *P-value < 0.05.

### Variable selection

**FIGURE 1:**
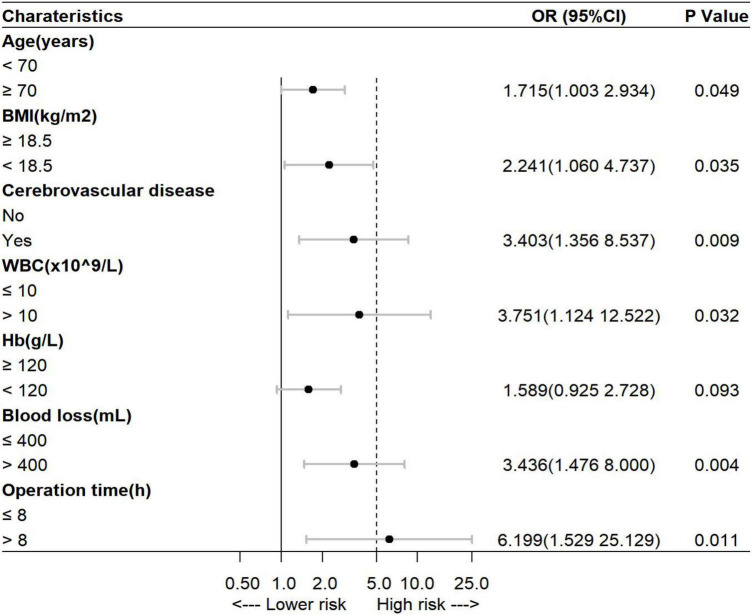
Multivariate analysis of the risk of postoperative cognitive dysfunction (POCD) in elderly patients undergoing gastrointestinal tumor resection. BMI, body mass index; WBC, white blood cell; Hb, hemoglobin.

The factors reported in previous literature that may affect the development of POCD–the level of education and the variables with *p* < 0.05 in the univariate analysis were subjected to multivariate logistic regression analysis with a stepwise elimination procedure to obtain an optimized model in terms of a minimal AIC value. Finally, age, BMI, history of cerebrovascular disease, preoperative WBC count, preoperative Hb level, intraoperative blood loss, and operation time were incorporated into the prediction model, and the minimum AIC value was 357.5. Among them, age ≥70 years [odds ratio (OR) = 1.715, 95% CI = 1.003–2.934], BMI <18.5 kg/m^2^ (OR = 2.241, 95% CI = 1.060–4.737), history of cerebrovascular disease (OR = 3.403, 95% CI = 1.356–8.537), preoperative WBC count >10 × 10^9^/L (OR = 3.751, 95% CI = 1.124–12.522), intraoperative blood loss >400 ml (OR = 3.436, 95% CI = 1.476–8.000), and operation time >8 h (OR = 6.199, 95% CI = 1.529–25.129) were independent risk factors for POCD in elderly patients with gastrointestinal tumors (*p* < 0.05, Figure 1). Variance inflation factors (VIFs) for variables included in the regression analyses were calculated to check for multicollinearity, which were all close to 1.0. It is indicated that there is no collinearity among seven variables (VIFs <5).

### Development of prediction model

**FIGURE 2:**
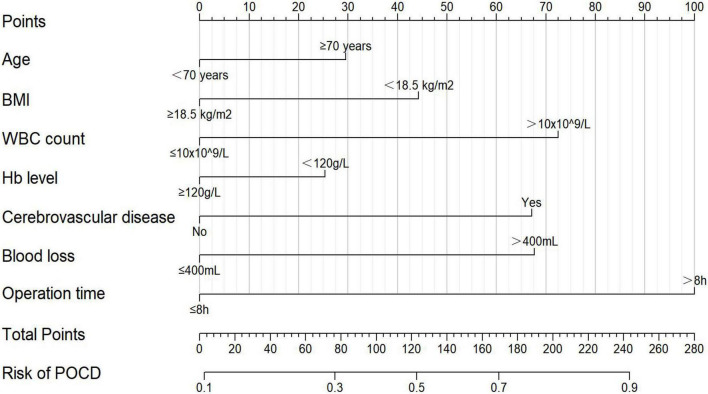
The nomogram for predicting the probability of POCD in elderly patients after gastrointestinal tumor resection. BMI, body mass index; WBC, white blood cell; Hb, hemoglobin.

According to the variables and their corresponding regression coefficients from the results of multivariate logistic regression analysis, the nomogram of POCD in elderly patients after gastrointestinal tumor resection was then established (Figure 2). Each of these variables is separately scored with a score ranging from 0 to 100. Furthermore, the scores for all variables were summed to generate the final total score, which corresponded to the probability of POCD in elderly patients undergoing gastrointestinal tumor resection (Figure 2).

### Performance of the postoperative cognitive dysfunction-risk nomogram and internal validation

**FIGURE 3:**
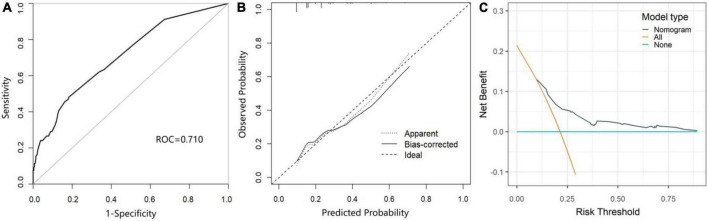
The performance and validation of the nomogram. **(A)** The receiver operating characteristic (ROC) curve of the prediction model for POCD. **(B)** The calibration curve of the nomogram-predicted probability of POCD. **(C)** Decision curve analysis (DCA) for the nomogram.

As shown in Figure 3, the area under the receiver operating characteristic curve (AUROC) of the predictive nomogram was 0.710 (95% CI = 0.645–0.775) (Figure 3A). Then, we performed the bootstrap method repeated sampling 1,000 times, and determined the internal validation of the nomogram model, with an AUROC of 0.709. In addition, the calibration curve showed that the predicted result of the nomogram model was in good agreement with the actual observed result, with a mean absolute error of 0.029 (Figure 3B). Moreover, the nomogram also performed well on the goodness of fit (Hosmer–Lemeshow test, χ^2^ = 5.133, *p* = 0.274). The decision curves indicated that when the threshold probability is above 0.1, the nomogram achieved more benefit than both the treat-all policy and the treat-none policy (Figure 3C).

## Discussion

In this study, we constructed a nomogram model for the prediction of POCD in elderly patients undergoing gastrointestinal tumor resection based on seven easily accessible items (age, history of cerebrovascular disease, BMI, preoperative WBC count, preoperative Hb level, intraoperative blood loss, and operation time). The performance of the nomogram was well demonstrated by the ROC curve, the calibration curve, and DCA in terms of discrimination, calibration plot, and clinical utility. To our knowledge, this is the first study to use a nomogram model to predict POCD in elderly patients undergoing gastrointestinal tumor resection. The benefit of such a model is the simultaneous realization of visualization as well as the objectification of measurement, which can be applied conveniently for clinical application.

The incidence of POCD was confirmed in 21.4% of patients in our study (79/369), whereas it was observed in 25.8% of patients in the International POCD Research Collaborative Group (Moller et al., 1998). The underlying reasons for the observed variability in incidence are differences between patient populations, the proportion of elderly people aged ≥70 years, and the diagnostic criteria of POCD. At the same time, our research showed that an age ≥70 years, a BMI <18.5 kg/m^2^, a history of cerebrovascular disease, a preoperative WBC count >10 × 10^9^/L, intraoperative blood loss >400 ml, and an operation time >8 h were risk factors of POCD in elderly patients undergoing gastrointestinal tumor resection.

The relationship between age and POCD is consistently reported in the literature, with numerous studies that implicate advanced age as a risk factor for POCD (Monk et al., 2008; O’Brien et al., 2017). In the process of aging, the structure and function of the brain change, such as a decrease in the overall weight of the brain, the volume of the corresponding brain regions, and the number of neurons and gliosis, while an increase in the permeability of the blood–brain barrier (Mattson and Magnus, 2006; Small et al., 2011; Elobeid et al., 2016; Daniele et al., 2018). All of these changes reduce the cerebral functional reserve and increase the risk of POCD. At the same time, our study showed that the frequency of POCD had a tendency to increase with a history of cerebrovascular disease, which was similar to previous studies (Monk et al., 2008). Evered et al. (2016) found that patients with a history of stroke were more likely to develop POCD even if they had no sequelae. Furthermore, the risk may also be raised even in asymptomatic patients who show neurovascular changes on brain imaging (white matter hyperintensity and cerebral infarction) (Kant et al., 2017).

A large prospective cohort study demonstrated that substantial weight loss (>10%) during follow-up was significantly associated with cognitive impairment (Wu et al., 2021), as confirmed by several other cohort studies (Driscoll et al., 2011; Ren et al., 2021). Similarly, we found that patients with a BMI <18.5 kg/m^2^ had a 1.241-fold increased risk of POCD. The mechanisms may be related to systemic inflammation, impaired metabolic function, gut microbiota (Grillner et al., 2016), and sarcopenia (Cruz-Jentoft et al., 2010; van Dam et al., 2018). In contrast, there were also other studies showing that elderly patients with a high BMI (>30 kg/m^2^) had an increased risk of cognitive impairment (Pérez-Belmonte et al., 2015; Feinkohl et al., 2016). This may be due to the general weight loss of patients with gastrointestinal tumors. All of the above findings suggested that an abnormal BMI may be an unfavorable factor for the maintenance of good cognition.

It is now generally believed that inflammation plays a substantial role in the pathogenesis of POCD (Skvarc et al., 2018). Peripheral inflammation is able to alter the structural and functional integrity of the blood–brain barrier, resulting in the loss of synaptic plasticity and neuronal apoptosis, which is detrimental to the recovery of postoperative brain function (Jin et al., 2020). In accordance with this, we detected that increased preoperative WBC counts were a risk factor for POCD. In addition, it has been reported that even within the normal range, increases in WBC counts are significantly associated with cognitive impairment (Kao et al., 2011; An et al., 2019).

In addition, our results showed that excessive intraoperative blood loss and prolonged operation time increased the risk of POCD in patients. As reported in animal experiments, acute blood loss caused postoperative visual-spatial memory and learning impairments in chronic hypertensive rats (Li et al., 2010). This may be due to excessive blood loss leading to intraoperative hypotension and ischemic damage to brain tissue, which in turn affects brain function (Fan et al., 2016; Hussein et al., 2019). Surgical trauma stimulation and a prolonged operation time would aggravate the damage caused by an insufficient cerebral blood supply, thereby affecting postoperative memory and cognitive function. Furthermore, clinical studies indicated that intraoperative blood loss, related to a systemic inflammatory status and blood-brain barrier disruption, may promote the postoperative brain (Taylor et al., 2022).

Postoperative cognitive dysfunction significantly affects the long-term outcomes and prognosis of patients. It is particularly important to identify high-risk groups and make great efforts to intervene. The nomogram, compared with computationally complex and tedious mathematical models, combines numerical probabilities with clinically meaningful variables to predict the risk of adverse events. It is a visual statistical formula model with seven simple indicators which are easy to obtain. Conveniently, the model helped clinical medical staff to identify patients who are prone to develop POCD early and accurately, which was of positive clinical guiding significance for carrying out targeted prevention strategies for POCD. According to our findings, we can reduce the occurrence as well as improve the prognosis of POCD by taking clinical measures, such as performing comprehensive preoperative assessments, improving patient nutritional status, actively correcting the inflammatory state, and adjusting surgical plans.

There are still some limitations to our study. First, our study was conducted in a single center with a limited number of patients. Moreover, it was performed among a specific population of patients with gastrointestinal tumors. However, since our model was based on seven easily available clinical indicators of patients with any surgical type, it could be verified in other surgical populations in further studies. In addition, our model still needs to be further improved. It is partly due to the fact that this was a retrospective study so some potentially associated factors of POCD reported in previous studies were not available, such as the frailty index, quality of life, and depth of anesthesia. Therefore, it is necessary to carry out prospective research, add relevant indicators to improve the prediction ability of the model and verify it in multiple centers.

## Data availability statement

The raw data supporting the conclusions of this article will be made available by the authors, without undue reservation.

## Ethics statement

The studies involving human participants were reviewed and approved by IRB of The Third Xiangya Hospital of Central South University. Written informed consent for participation was not required for this study in accordance with the national legislation and the institutional requirements.

## Author contributions

HH, XW, and YL conceived and designed the study. HH and JC performed the statistical analysis and wrote the first draft of the manuscript. YT contributed to the manuscript revision. WO, XW, and YL contributed to the writing of the final version of the manuscript. All authors contributed to the article and approved the submitted version.

## References

[B1] Alam A. Hana Z. Jin Z. Suen K. C. Ma D. (2018). Surgery, neuroinflammation and cognitive impairment. *EBioMedicine* 37 547–556. 10.1016/j.ebiom.2018.10.021 30348620 PMC6284418

[B2] An P. Zhou X. Du Y. Zhao J. Song A. Liu H. (2019). Association of neutrophil-lymphocyte ratio with mild cognitive impairment in elderly chinese adults: A case-control study. *Curr. Alzheimer Res.* 16 1309–1315. 10.2174/1567205017666200103110521 31902361

[B3] Boone M. D. Sites B. von Recklinghausen F. M. Mueller A. Taenzer A. H. Shaefi S. (2020). Economic burden of postoperative neurocognitive disorders among us medicare patients. *JAMA Netw. Open* 3:e208931. 10.1001/jamanetworkopen.2020.8931 32735336 PMC7395237

[B4] Borsook D. George E. Kussman B. Becerra L. (2010). Anesthesia and perioperative stress: Consequences on neural networks and postoperative behaviors. *Prog. Neurobiol.* 92 601–612. 10.1016/j.pneurobio.2010.08.006 20727935

[B5] Cao L. J. Dong H. L. Fang X. M. Wang X. P. Liu K. X. Gu X. P. (2018). Ten scientific problems to be solved urgently in anesthesiology. *Chin. J. Anesthesiol.* 38 4–7.

[B6] Cruz-Jentoft A. J. Baeyens J. P. Bauer J. M. Boirie Y. Cederholm T. Landi F. (2010). Sarcopenia: European consensus on definition and diagnosis: Report of the European working group on sarcopenia in older people. *Age Ageing* 39 412–423. 10.1093/ageing/afq034 20392703 PMC2886201

[B7] Daniele S. Giacomelli C. Martini C. (2018). Brain ageing and neurodegenerative disease: The role of cellular waste management. *Biochem. Pharmacol.* 158 207–216. 10.1016/j.bcp.2018.10.030 30393045

[B8] Driscoll I. Espeland M. A. Wassertheil-Smoller S. Gaussoin S. A. Ding J. Granek I. A. (2011). Weight change and cognitive function: Findings from the Women’s Health Initiative Study of Cognitive Aging. *Obesity (Silver Spring).* 19 1595–1600. 10.1038/oby.2011.23 21394095 PMC3175491

[B9] Duan S. Liao Y. Tang Y. Zhang B. Peng M. Tong J. (2022). Short-term perioperative cognitive therapy combined with rehabilitation exercise reduces the incidence of neurocognitive disorder in elderly patients: A randomized controlled trial. *Minerva Anestesiol.* 88 145–155. 10.23736/s0375-9393.21.15877-8 35315627

[B10] Eckenhoff R. G. Maze M. Xie Z. Culley D. J. Goodlin S. J. Zuo Z. (2020). Perioperative neurocognitive disorder: State of the preclinical science. *Anesthesiology* 132 55–68. 10.1097/aln.0000000000002956 31834869 PMC6913778

[B11] Elobeid A. Libard S. Leino M. Popova S. N. Alafuzoff I. (2016). Altered proteins in the aging brain. *J. Neuropathol. Exp. Neurol.* 75 316–325. 10.1093/jnen/nlw002 26979082 PMC4793886

[B12] Evered L. A. Chan M. T. V. Han R. Chu M. H. M. Cheng B. P. Scott D. A. (2021). Anaesthetic depth and delirium after major surgery: A randomised clinical trial. *Br. J. Anaesth.* 127 704–712. 10.1016/j.bja.2021.07.021 34465469 PMC8579421

[B13] Evered L. A. Silbert B. S. Scott D. A. Maruff P. Ames D. (2016). Prevalence of dementia 7.5 years after coronary artery bypass graft surgery. *Anesthesiology* 125 62–71. 10.1097/aln.0000000000001143 27127919

[B14] Fan D. Li J. Zheng B. Hua L. Zuo Z. (2016). Enriched environment attenuates surgery-induced impairment of learning, memory, and neurogenesis possibly by preserving bdnf expression. *Mol. Neurobiol.* 53 344–354. 10.1007/s12035-014-9013-1 25432890

[B15] Feinkohl I. Winterer G. Pischon T. (2016). Obesity and post-operative cognitive dysfunction: A systematic review and meta-analysis. *Diabetes Metab. Res. Rev.* 32 643–651. 10.1002/dmrr.2786 26890984

[B16] Grillner S. Ip N. Koch C. Koroshetz W. Okano H. Polachek M. (2016). Worldwide initiatives to advance brain research. *Nat. Neurosci.* 19 1118–1122. 10.1038/nn.4371 27571190 PMC6047900

[B17] Gvozdenović L. Antanasković A. (2015). History of alcohol abuse after major non-cardiac surgery and postoperative cognitive dysfunction. *Eur. J. Intern. Med.* 26:e51. 10.1016/j.ejim.2015.07.001 26198786

[B18] Hussein M. Fathy W. Nabil T. Abd Elkareem R. (2019). Postoperative cognitive dysfunction and the possible underlying neurodegenerative effect of anaesthesia. *Int. J. Neurosci.* 129 729–737. 10.1080/00207454.2018.1561451 30590973

[B19] Jin Z. Hu J. Ma D. (2020). Postoperative delirium: Perioperative assessment, risk reduction, and management. *Br. J. Anaesth.* 125 492–504. 10.1016/j.bja.2020.06.063 32798069

[B20] Kant I. M. J. de Bresser J. van Montfort S. J. T. Slooter A. J. C. Hendrikse J. (2017). MRI markers of neurodegenerative and neurovascular changes in relation to postoperative delirium and postoperative cognitive decline. *Am. J. Geriatr. Psychiatry* 25 1048–1061. 10.1016/j.jagp.2017.06.016 28760515

[B21] Kao T. W. Chang Y. W. Chou C. C. Hu J. Yu Y. H. Kuo H. K. (2011). White blood cell count and psychomotor cognitive performance in the elderly. *Eur. J. Clin. Invest.* 41 513–520. 10.1111/j.1365-2362.2010.02438.x 21466549

[B22] Kotekar N. Shenkar A. Nagaraj R. (2018). Postoperative cognitive dysfunction - current preventive strategies. *Clin. Interv. Aging* 13 2267–2273. 10.2147/cia.S133896 30519008 PMC6233864

[B23] Li M. Bertout J. A. Ratcliffe S. J. Eckenhoff M. F. Simon M. C. Floyd T. F. (2010). Acute anemia elicits cognitive dysfunction and evidence of cerebral cellular hypoxia in older rats with systemic hypertension. *Anesthesiology* 113 845–858. 10.1097/ALN.0b013e3181eaaef9 20808217 PMC3233697

[B24] Mahanna-Gabrielli E. Zhang K. Sieber F. E. Lin H. M. Liu X. Sewell M. (2020). Frailty is associated with postoperative delirium but not with postoperative cognitive decline in older noncardiac surgery patients. *Anesth. Analg.* 130 1516–1523. 10.1213/ane.0000000000004773 32384341 PMC7875454

[B25] Margraf A. Ludwig N. Zarbock A. Rossaint J. (2020). Systemic inflammatory response syndrome after surgery: Mechanisms and protection. *Anesth. Analg.* 131 1693–1707. 10.1213/ane.0000000000005175 33186158

[B26] Mattson M. P. Magnus T. (2006). Ageing and neuronal vulnerability. *Nat. Rev. Neurosci.* 7 278–294. 10.1038/nrn1886 16552414 PMC3710114

[B27] Moller J. T. Cluitmans P. Rasmussen L. S. Houx P. Rasmussen H. Canet J. (1998). Long-term postoperative cognitive dysfunction in the elderly ISPOCD1 study. ISPOCD investigators. international study of post-operative cognitive dysfunction. *Lancet* 351 857–861. 10.1016/s0140-6736(97)07382-0 9525362

[B28] Monk T. G. Weldon B. C. Garvan C. W. Dede D. E. Aa M. T. Heilman K. M. (2008). Predictors of cognitive dysfunction after major noncardiac surgery. *Anesthesiology* 108 18–30. 10.1097/01.anes.0000296071.19434.1e 18156878

[B29] Murthy S. Hepner D. L. Cooper Z. Bader A. M. Neuman M. D. (2015). Controversies in anaesthesia for noncardiac surgery in older adults. *Br. J. Anaesth.* 115(Suppl. 2) ii15–ii25. 10.1093/bja/aev396 26658197 PMC4809989

[B30] Needham M. J. Webb C. E. Bryden D. C. (2017). Postoperative cognitive dysfunction and dementia: What we need to know and do. *Br. J. Anaesth.* 119 i115–i125. 10.1093/bja/aex354 29161395

[B31] Newman S. Stygall J. Hirani S. Shaefi S. Maze M. (2007). Postoperative cognitive dysfunction after noncardiac surgery: A systematic review. *Anesthesiology* 106 572–590. 10.1097/00000542-200703000-00023 17325517

[B32] O’ Brien H. Mohan H. Hare C. O. Reynolds J. V. Kenny R. A. (2017). Mind over matter? the hidden epidemic of cognitive dysfunction in the older surgical patient. *Ann. Surg.* 265 677–691. 10.1097/sla.0000000000001900 27537541

[B33] Peden C. J. Miller T. R. Deiner S. G. Eckenhoff R. G. Fleisher L. A. (2021). Improving perioperative brain health: An expert consensus review of key actions for the perioperative care team. *Br. J. Anaesth.* 126 423–432. 10.1016/j.bja.2020.10.037 33413977

[B34] Pérez-Belmonte L. M. San Román-Terán C. M. Jiménez-Navarro M. Barbancho M. A. García-Alberca J. M. Lara J. P. (2015). Assessment of long-term cognitive impairment after off-pump coronary-artery bypass grafting and related risk factors. *J. Am. Med. Dir. Assoc.* 16 .e9–.e263. 10.1016/j.jamda.2014.12.001 25648462

[B35] Ren Z. Li Y. Li X. Shi H. Zhao H. He M. (2021). Associations of body mass index, waist circumference and waist-to-height ratio with cognitive impairment among Chinese older adults: Based on the CLHLS. *J. Affect. Disord.* 295 463–470. 10.1016/j.jad.2021.08.093 34507227

[B36] Rengel K. F. Mehdiratta N. Vanston S. W. Archer K. R. Jackson J. C. Thompson J. L. (2021). A randomised pilot trial of combined cognitive and physical exercise prehabilitation to improve outcomes in surgical patients. *Br. J. Anaesth.* 126 e55–e57. 10.1016/j.bja.2020.11.004 33317805 PMC8040115

[B37] Rundshagen I. (2014). Postoperative cognitive dysfunction. *Dtsch. Arztebl. Int.* 111 119–125. 10.3238/arztebl.2014.0119 24622758 PMC3959222

[B38] Skvarc D. R. Berk M. Byrne L. K. Dean O. M. Dodd S. Lewis M. (2018). Post-operative cognitive dysfunction: An exploration of the inflammatory hypothesis and novel therapies. *Neurosci. Biobehav. Rev.* 84 116–133. 10.1016/j.neubiorev.2017.11.011 29180259

[B39] Small S. A. Schobel S. A. Buxton R. B. Witter M. P. Barnes C. A. (2011). A pathophysiological framework of hippocampal dysfunction in ageing and disease. *Nat. Rev. Neurosci.* 12 585–601. 10.1038/nrn3085 21897434 PMC3312472

[B40] Su X. Meng Z. T. Wu X. H. Cui F. Li H. L. Wang D. X. (2016). Dexmedetomidine for prevention of delirium in elderly patients after non-cardiac surgery: A randomised, double-blind, placebo-controlled trial. *Lancet* 388 1893–1902. 10.1016/s0140-6736(16)30580-3 27542303

[B41] Susano M. J. Grasfield R. H. Friese M. Rosner B. Crosby G. Bader A. M. (2020). Brief preoperative screening for frailty and cognitive impairment predicts delirium after spine surgery. *Anesthesiology* 133 1184–1191. 10.1097/aln.0000000000003523 32898243 PMC7657972

[B42] Taylor J. Parker M. Casey C. P. Tanabe S. Kunkel D. Rivera C. (2022). Postoperative delirium and changes in the blood-brain barrier, neuroinflammation, and cerebrospinal fluid lactate: A prospective cohort study. *Br. J. Anaesth.* 129 219–230. 10.1016/j.bja.2022.01.005 35144802 PMC9465948

[B43] United Nations: Department of Economic and Social Affairs (2021). *Ageing populations: We are living longer lives, but are we healthier?.* New York, NY: United Nations.

[B44] Urits I. Orhurhu V. Jones M. Hoyt D. Seats A. Viswanath O. (2019). Current perspectives on postoperative cognitive dysfunction in the ageing population. *Turk. J. Anaesthesiol. Reanim.* 47 439–447. 10.5152/tjar.2019.75299 31828240 PMC6886822

[B45] van Dam R. Van Ancum J. M. Verlaan S. Scheerman K. Meskers C. G. M. Maier A. B. (2018). Lower cognitive function in older patients with lower muscle strength and muscle mass. *Dement. Geriatr. Cogn. Disord.* 45 243–250. 10.1159/000486711 29913450 PMC6067649

[B46] Wang P. Yin X. Chen G. Li L. Le Y. Xie Z. (2021). Perioperative probiotic treatment decreased the incidence of postoperative cognitive impairment in elderly patients following non-cardiac surgery: A randomised double-blind and placebo-controlled trial. *Clin. Nutr.* 40 64–71. 10.1016/j.clnu.2020.05.001 32451125

[B47] Wu S. Lv X. Shen J. Chen H. Ma Y. Jin X. (2021). Association between body mass index, its change and cognitive impairment among Chinese older adults: A community-based, 9-year prospective cohort study. *Eur. J. Epidemiol.* 36 1043–1054. 10.1007/s10654-021-00792-y 34370136

[B48] Zietemann V. Georgakis M. K. Dondaine T. Müller C. Mendyk A. M. Kopczak A. (2018). Early MoCA predicts long-term cognitive and functional outcome and mortality after stroke. *Neurology* 91 e1838–e1850. 10.1212/wnl.0000000000006506 30333158

